# Do Safe Bike Lanes Really Slow Down Cars? A Simulation-Based Approach to Investigate the Effect of Retrofitting Safe Cycling Lanes on Vehicular Traffic

**DOI:** 10.3390/ijerph19073818

**Published:** 2022-03-23

**Authors:** Pivithuru Kalpana Nanayakkara, Nano Langenheim, Irene Moser, Marcus White

**Affiliations:** 1 Department of Computing Technologies, Swinburne University of Technology, John Street, Hawthorn, VIC 3122, Australia; imoser@swin.edu.au; 2 Melbourne School of Design, University of Melbourne, Masson Rd, Parkville, VIC 3010, Australia; nano.langenheim@unimelb.edu.au; 3 Department of Interior Architecture and Industrial Design, Swinburne University of Technology, John Street, Hawthorn, VIC 3122, Australia; marcuswhite@swin.edu.au

**Keywords:** safe cycling, street designs, bike lane, street reconfiguration, simulation-based modelling, traffic simulation, active transport, retrofitting sustainable transport

## Abstract

Cycling is a sustainable transportation mode that provides many health, economic and environmental benefits to society. Cities with high rates of cycling are better placed to address modern challenges of densification, carbon-neutral and connected 20-min neighbourhood goals. Despite the known benefits of cycling, participation rates in Australian cities are critically low and declining. Frequently, this low participation rate is attributed to the dangers of Australian cycle infrastructure that often necessitates the mixing of cyclists with car traffic. In addition, residents of car-dependent Australian suburbs can be resistant to the installation of cycle infrastructure where threats to traffic flow, or decreased on-street parking availability are perceived and the prohibitive cost of reconfiguration of other infrastructure maintained by the local councils to retrofit safe bike paths. This study investigates the effects on traffic behaviour of retrofitting safe, separate cycling lanes into existing residential streets in a Melbourne suburb suitable for accessing the primary neighbourhood destinations. We utilise only the widths available on the existing roadway of these streets, with minimal incursion on other facilities, such as the vehicle network and parking. Using only the existing roadway reflects the common need for municipal asset managers to minimise disruption and costs associated with street redesign. Using a traffic simulation approach, we modelled travel demand that suits suburban trips to services and shops, and we selectively applied separate cycling lanes to suitable residential streets and varied the effect of lowering speed limits. Simulations show that the selective inclusion of safe cycling lanes in some streets leads to a mere 7% increase in the average car travel times in the worst case, while requiring cyclists to increase their travel distance only marginally to avoid streets without dedicated cycling lanes. These results demonstrate that reasonable compromises are possible to make suburbs safer for cyclists and bring them closer to the 20-min neighbourhood goal. There is significant potential to enhance the result by including more street types and alternative designs. The results can inform councils in their cycle path infrastructure decisions and disprove assumptions about the influence of cyclists on car infrastructure.

## 1. Introduction

Cycling is a sustainable transportation mode that provides many health, economic and environmental benefits to society. Yet, in many developed countries, such as Australia, Canada and the United States, private motorised vehicles are still the dominant transportation mode [1]. The implementation of safe cycle infrastructure prioritised by many European countries over the past half-century has resulted in significantly higher rates of cycling participation in those cities. For example, bicycle modal share is only 1% of daily trips in Australia and the United States, while in the Netherlands, it constitutes 27% of trips [2,3].

Although many major Australian cities including Melbourne, Sydney and Brisbane have cycling infrastructure and populations who value physical fitness, increasing or even maintaining the rate of cycling in these cities has been difficult to achieve [4]. This is also despite cities such as Melbourne having a temperate climate with relatively desirable weather conditions for cycling comparable to the Netherlands or Northern Germany [5]. As a nation, 69% of the Australian working population commute to work by car and only 1.1% cycle [6]. While cycling is unpopular for commuting in Australia, the biannual National Cycling Participation Surveys (NCPS) consistently show that it is popular for recreational trips. In 2019, the survey showed only 31.7% of participants rode for transport, while 81.9%, rode for recreational purposes [7]. These figures highlight the willingness of Australian citizens to cycle on recreational bike trails where dedicated safe cycle pathways are readily available, but there are barriers to cycling as a mode of transport for road-based trips. Research into the barriers to road-based cycling in Australia consistently point to both lack of cycle path continuity and a lack of separation from adjacent traffic [8,9].

Cycling lanes that provide a minimum of 1 m width on flat terrain are separated from traffic with provision of a physical curb buffer or barrier between vehicular traffic and cyclists, and increase both real and perceived cyclist safety and promote cycling as a mode of transport. An example of real safety offered by separated cycling paths was investigated by Nolan et al. which found that Australian motorists keep a larger distance from dedicated cycling lanes than they do from cyclists in mixed traffic, even when controlling for the space available on the roadway, and protected (raised physical curb barrier) bicycle lanes completely remove the risk of ‘passing events’ less than 1 m [8]. In another study by Schepers et al., the findings show that separate, buffered cycling lanes on Utrecht’s bicycle path network not only contribute the highest rate of bicycle modal share, but also offer the greatest perceived cyclist safety [9].

While wider cycle paths are preferable and can reduce risk of ‘passing events’ as much as 4% when lanes are increased from 1 m to 2.5 m [8], for the purposes of this study, we have taken ‘safe cycle lanes’ to refer to lanes that are dedicated, separated by a physical curb barrier, and have a minimum of 1 m width as stipulated in current Austroads design guidelines [10].

**Figure 1:**
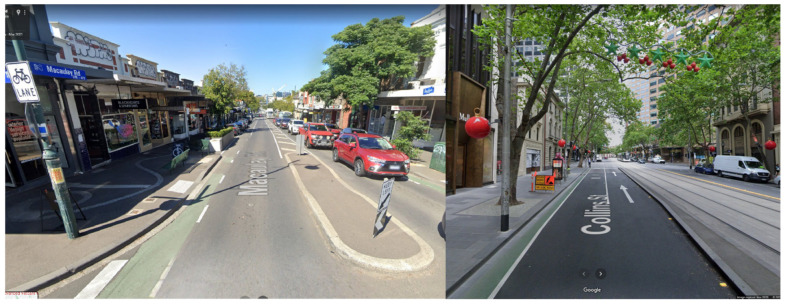
Google Street View screengrabs showing examples of cycle lanes being ‘squeezed in’ within the City of Melbourne cycle lanes that are narrow (less than 0.6 m wide) and do not provide physical separation by a concrete curb. (**left**) Kensington and (**right**) Collins Street Melbourne.

To retrofit cycle lanes into existing road networks is challenging and can require what Steele and McTiernan referred to as “squeezing them in” [11]. An illustration of this challenge can be seen in examples from the local government of the City of Melbourne (Figure 1), where cycle lanes have been ‘squeezed’ to be an impractical width and provide little separation from cars. While the ideal recommended, one-way bike lane width is between 1.5 to 2.5 m, to retrofit physically separated cycle lanes of these dimensions is unrealistic in much of the Australian context for two primary reasons [12]. Firstly, roads represent the single most expensive asset maintained by local governments and contain several other concurrent infrastructure components such as drainage lines and services. Reconfiguring this infrastructure on a broad scale can represent prohibitive public cost [13,14]. Secondly, local governments are required to balance infrastructure planning with the voice of residents. High levels of car ownership in Australia mean sensitivity to both removal of on-street car parking privileges and negative impacts to already long motor vehicle commute times [15,16].

Many Australian streets are, however, quite wide, but traffic regulations dedicate most of that available space to comparatively wide vehicle lane widths (3.25 m–3.6 m) compared to other European and Asia Pacific countries (2.8 m–3.25 m) [17]. Post 1950, Australian streets were generally developed for wider cars with little regard for cycling and walking [18]. This street space could instead be reconfigured to provide more continuous, traffic separated, safe cycling networks, however, these local traffic regulations and resident resistance barriers must be overcome. Support for any changes made to the street configuration must be gained from local communities who own and use motor vehicles, and common street layouts need to be adjusted to favour narrower vehicle lane widths.

To address these barriers to street reconfiguration to increase safe cycling in Australian cities, this study aims to answer two questions, “Can the roadways of local streets be reconfigured to include safe cycling lanes?” and if so, “do safe bike lanes on streets really slow down cars?”

To find an answer to these questions, we used a two-step process of network reconfiguration, making adjustments to enable a safe cycling network, without major disruption to existing road infrastructure, or loss of car parking spaces, and then using a traffic simulator to simulate cyclists and cars in the road network, observing the effects of the cycling network on motor vehicle-based users.

This paper is organised into nine main sections and begins with the background of barriers to uptake cycling. The next section presents the method of redesigning local streets with cycling lanes to optimise vehicular traffic in a portion of a residential area followed by the evaluation of the road network models used for traffic simulations. The next two sections provide the results of experiments and discussion followed by the threats to research validity and future work. The research conclusions are outlined in the final section.

## 2. Background

### 2.1. Cycling for Human and Environmental Health

Cycling is a reliable and affordable transportation option for shorter distance trips that helps to reduce automobile traffic, carbon emissions and noise pollution [19]. Increasing rates of cycling has become an increasingly important proponent in global efforts to mitigate the impact of climate change and transition towards more sustainable, higher density development models, particularly “20-min neighbourhoods” [20,21,22]. Cycling provides many health benefits associated with physical activity, such as reduction in premature deaths, heart disease, cancer risk and obesity [23,24,25,26,27,28,29,30]. While there are studies on cycling that show it is associated with increased risk of traffic injuries, fatalities and exposure to polluted air [31,32], the health benefits of cycling are demonstrated to considerably outweigh these risks, thus supporting the argument for concentrated efforts toward increasing cycling uptake while also ensuring high standards of cyclist safety [33,34].

### 2.2. Impact of Safe Cycling Infrastructure

The traffic fatality statistics from the Organization for Economic Cooperation and Development (OECD) report a reduction in cycling mortality by 30% in the USA, 46% in Australia and 49% in Canada from 1990 to 2014 as a result of improved cycling safety. The measures taken included implementing exclusive bike lanes with physical curb separators, providing off-road cycling tracks and reducing adjacent traffic speeds [35]. Converting the outer lane into a dedicated cycling lane has been found to improve cycling safety in another study [36].

### 2.3. Reduced Speed and Physical Separation

Globally, literature on traffic safety finds correlations between higher traffic speeds and collision rates [37]. A recent Australian study showed that reducing the default speed limit from 60 km/h to 50 km/h throughout Melbourne in the past decade, has led to a 12% decrease in casualties and a 25–40% reduction in fatalities in Victoria from 2001 to 2003 [38]. In addition, the World Resource Institute (WRI) reported that speed limits of 50 km/h, 40 km/h and 30 km/h have, respectively, 85%, 30% and 10% probability of fatal accidents involving cyclists [39]. Reducing traffic speed limits on streets would significantly improve safety for cyclists.

However, reducing vehicle speed limits alone does not necessarily make a safe cycling environment. The most commonly reported road traffic injury for cyclists is car dooring crashes, associated with poor separation of cyclists from adjacent vehicle traffic [31,40]. VicRoads reported 726 car dooring crashes between 2014 and 2019 [41]. Building a safe cycling infrastructure, i.e., physically separated and protected cycling lanes, increases the safety of all road users, cyclists and drivers alike [42]. Physical separators between cycling and adjacent vehicle traffic lanes enhance the actual and perceived safety of cyclists and promote cycling uptake [43]. A study conducted in Australia found that there is no risk of passing vehicles less than 1 m closer to cyclists where bike lanes are protected with a physical barrier such as a concrete curb, compared to the other bicycle lane types, for example, dotted line, painted and wide painted buffer bike lanes [8].

### 2.4. Australian Context–Citizens’ Resistance to Cycle-Friendly Change Due to Fear of Cost, Loss of Parking and Increase of Congestion

Currently, in Australian suburbs, the dominant configuration of existing streets dedicate the roadway to vehicle traffic lanes and parking. The adjacent verge is separated from the roadway by the kerb on either side. The street verge accommodates infrastructure such as utilities (electricity, water supply, stormwater drainage, sewerage, gas, telephone and internet) a footpath, and a nature strip. Any disruptions to the current configuration, particularly the kerb location, can have profound financial implications due to the potential disruption of these underground services.

Much of the development of Australia was concurrent with the development of the motor vehicle [44]. It is difficult to change travel behaviour in sprawling Australian suburbs as distances between primary destinations are much larger than in European cities, public transport provision is poor and individual rates of car ownership are high. Local councils thus face challenges in suggesting removal of parking lanes and reduction of traffic lane widths to create space for cycling lanes amongst local communities who predominantly use personal motor vehicles for transport [45]. Residents are generally opposed to losing on-street parking, and there is a perception of slower progress on narrower streets [46,47].

A recent study by Wild et al. [48] on the ‘Bikelash’ phenomenon uncovers the hostility communities harbour towards cycling lanes, capturing the anger motorists exhibit when cycling lanes reduce car parking. Car owners see their rights extending to on-street parking privileges, the removal of which is likely to be met with strong local community resistance [16]. An analysis of claims made by residents in Melbourne demonstrates the imbalance in the planning system and pressure to create more parking spaces [49].

The ‘Bikelash’ also extends to anger over potential congestion or being ‘slowed down’ by either reduced speed limits, or the concern for losing lane width space to bike lanes [48]. Developments that are perceived to potentially ‘worsen congestion’ are met with extremely strong resident resistance [50]. In addition, reduction of speed limits in residential streets have been met with considerable opposition from the community at large, who believe lower speed limits will impose time costs on motorists [51].

The anti-cycle lane sentiment relating to concerns for car parking, time cost or additional congestion can translate into powerful organised opposition to bike lanes: people coming together to form groups and develop campaigns to have lanes removed [48].

It is therefore important when proposing retrofitting cycling lanes into existing Australian suburban streets, to consider the aspects of likely community resistance to street design changes, the challenging realities of implementation, and to focus on incremental change where major changes are not feasible [52] (p. 9).

### 2.5. Agent-Based Modelling

In previous studies, the Agent-Based Modelling (ABM) approach was used to simulate cycling traffic to analyse route choice decisions of cyclists, travel speed and time by considering cycling infrastructure and related attributes including surface and gradient [53,54].

Wallentin and Loidl [55] used the ABM to observe the spatial distribution of cyclists in the inner city and outer city of Salzburg by simulating cycling traffic. The impact on cyclists’ safety was explored using ABM by incorporating the behaviour of drivers and a high level of continuously dedicated cycling infrastructure in the network [56]. The current study also employs the ABM approach to simulate mixed traffic conditions in a residential area to observe the network efficiency in terms of the average speed of agents (cars) in the network with and without dedicated cycling lanes. The baseline network model and test scenarios were developed to explore the status quo and future traffic conditions with cycling infrastructure, respectively. These agents in the network take short trips from their residences to services within the network. These origin destination (OD) trips generate the traffic demand within the network during simulations. The collection of OD trips (Demand Matrix) by car and bicycle are input into the traffic simulation software and set the speed limits of local streets for the baseline model and future scenarios. The simulation model allows to test the impact on average speeds of agents after application of cycling lanes to existing local streets and adjusting speed limits in local streets to reflect the modifications of existing street space.

## 3. Reconfiguring Street Space to Include Safe Cycling

In this section, we provide the method used for this study to improve Australian local streets by adding safe bike lanes. As the initial step, we redesigned street layouts with safe cycling lanes. The next section provides details on the selective application of street designs for suitable street width ranges followed by the creating of local traffic demand and modelling road network for traffic simulation.

### 3.1. The Approach of This Study to Street Redesign

This study proposes an approach for minimal intervention modification to existing street space for the purpose of adding safe, separate cycling lanes by devising new street layouts that minimise the impact on network efficiency. These new design options could be used to optimise local street space to foster multi-modal transportation. In this study, we employ the traffic simulation to demonstrate the implications for network efficiency of the application of safe cycling infrastructure.

### 3.2. Design of Street Layouts for Cycling

This study focuses on the purposeful redesign of local streets using existing street space to include safe cycling. According to the Austroads Guide to Road Design, the typical urban local street roadway width is 10.8 m [12]. However, depending on the era of suburb development, within and across Australia, local streets vary in width ranging from between 5 m to 14 m [57]. These local streets are heterogeneously configured according to the available space and generally do not include dedicated cycling lanes.

The most prevalent local residential street configuration we find in Australian suburbs are between 10 m and 14 m wide, with two central traffic lanes in opposite directions, and parking lanes, nature strips and footpaths on each side [12]. While these streets have space for several configurations that include safe, separate cycling spaces, cyclists are currently weaving around parked vehicles, and forced to mix with vehicular traffic, which impacts the speed and safety of both cycling and car movement.

Local streets in Australian suburbs are often organised in a cardinally oriented rectangular lattice, with general roadway (kerb to kerb) widths between 5 m to 14 m in residential areas and up to 18 m in industrial areas. While in Melbourne, most local streets have a 50 km/h speed limit, which prior to 2001 had a speed limit of 60 km/h [58], just as some local streets in the Northern Territory do [10]. These streets could be reconfigured to accommodate safe cycling lanes. To avoid the cost of moving gutter/kerb and stormwater services, the design modifications are kept within the existing roadway.

While removing parking space on local streets is a cost-effective way of including cycling lanes, for this study we did not design street layouts by eliminating parking lanes. Instead, space for bike lanes was obtained from the wide car traffic lanes (see example street Figure 1). However, in many streets, the narrowing of traffic lanes would require speed limit reductions, which are also generally unpopular. It is therefore helpful to provide evidence that changing the street network will not dramatically reduce network efficiency.

In this study, we are looking at a subset of streets that are between 10 m and 12 m wide in order to include safe cycling lanes. A large proportion of local streets in Australia fall into this category. The common existing design layout for these streets consists of two traffic lanes in opposite directions and parking lanes on both sides. Therefore, in this study, we consider the most predominant street width category and its potential to accommodate cycling lanes. We divided this subset of streets into two width ranges, each suitable for a group of cycling-inclusive layouts. Type 1 represents 10.5 m to 11.5 m wide residential streets and provides limited space for narrow cycling lanes, while Type 2 comprises streets with a width of 11.5 m or above and can accommodate more generous cycling spaces. For each type, we devised suitable variations of cycling-inclusive layouts.

**Figure 2:**
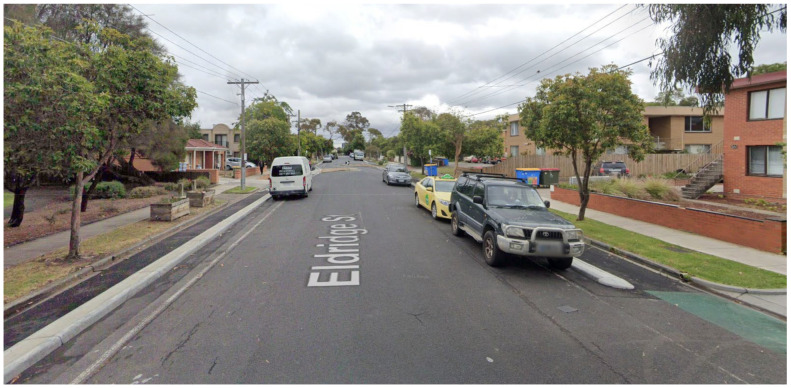
Google Street View screengrab of Eldridge Street Footscray, in Melbourne’s inner west, showing recent retrofit of cycling lanes onto an existing residential street to include 300 mm physical barrier curb separation with 800 mm cycle path.

To include cycling lanes, road elements are reduced in width, and one-way or two-way cycling lanes are added according to the recommendations of the Cycling Aspects of Austroads Guide [10]. The new designs include a minimum 0.3 m wide concrete barrier, which serves as the physical separator between the bike lane and parking lane and helps reduce the risk of ‘dooring’, similar to the example street shown in Figure 2.

**Figure 3:**
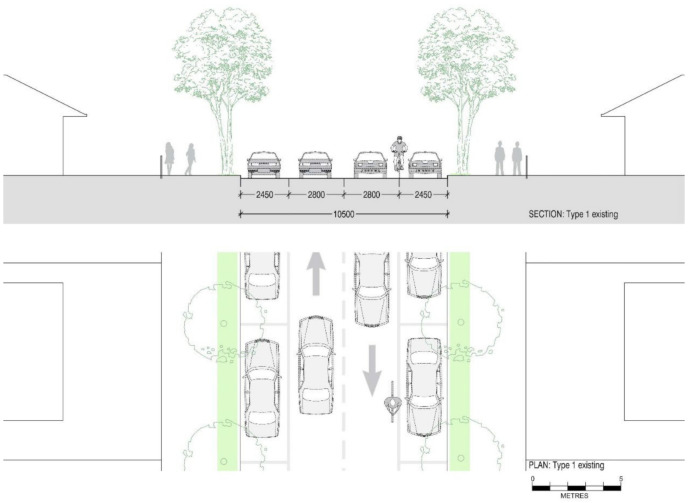
Section and plan drawings showing ‘Type 1′ existing street configuration with between 10.5 m and 11.5 m street roadway width, 2.45 m parking spaces on either side of the streets and two-way single traffic lanes of 2.6 m width to accommodate 50–60 km/h s speed limit.

Figure 3 shows the typical layout of a street we defined as Type 1. These streets provide a generous 2.8 m width for traffic lanes, designed for the speed limits of 60 km/h applied in the past (now 50 km/h) and 2.45 m wide parking. If speed limits are reduced to 40 km/h, according to Austroads Guide to Road Design [59], a lane width of 2.5 m is sufficient, leaving space for the inclusion of separate cycling lanes. According to Austroads, parking spaces only require a width of 2.1 m, leaving 1.3 m for the inclusion of a cycling lane without reducing the current facilities. This is sufficient for the inclusion of a 1000 mm one-directional cycling lane with a 300 mm separation from car and parking lanes.

**Figure 4:**
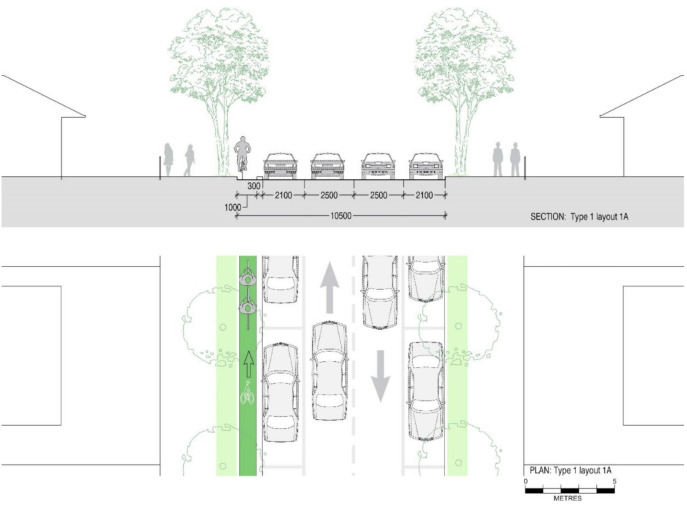
Section and plan drawings showing proposed retrofit ‘Design 1A’ for a reconfigured ‘Type 1’ street including a 1 m single cycling lane and 0.3 m wide physical barrier with similar roadway, but 2.1 m parking lanes on either side of the streets and two-way single traffic lanes of 2.5 m width to accommodate 40 km/h speed limit.

**Figure 5:**
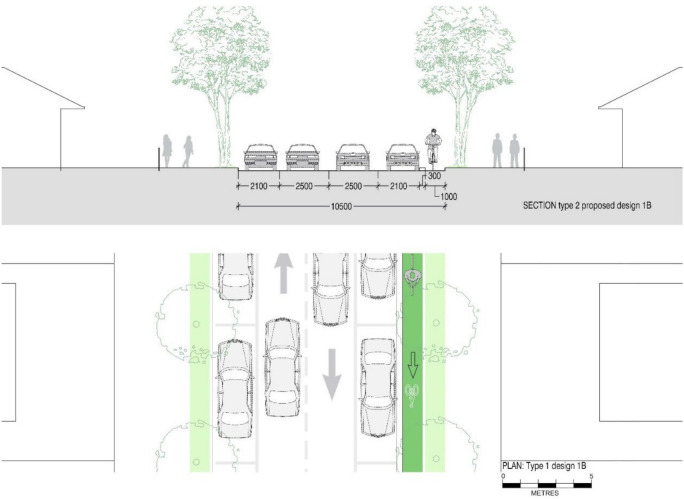
Section and plan drawings showing proposed retrofit ‘Design 1B’ for opposite direction of a reconfigured ‘Type 1’ street including a 1 m single cycling lane and 0.3 m wide physical barrier with similar roadway, but 2.1 m parking lanes on either side of the streets and two-way single traffic lanes of 2.5 m width to accommodate 40 km/h speed limit.

The road elements of the new design contain a one-directional cycling lane, barrier, two traffic lanes in opposite directions and two parking lanes. This reconfiguration allows cyclists to ride safely in a single direction. This design has to be matched with a corresponding road that includes a cycling lane in the opposite direction. Design 1A contains a one-way cycling lane on the left side of the street along with existing street elements in Type 1 streets. In contrast to design 1A, design 1B contains one-way cycling on the right side of the street to provide access to the opposite direction in the road network. Figure 4 and Figure 5 display new street designs (Design 1A and Design 1B) with a one-directional cycling lane for Type 1 streets.

**Figure 6:**
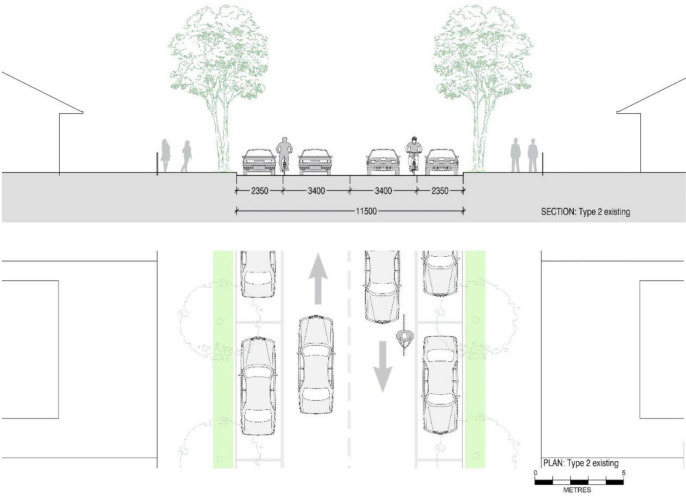
Section and plan drawings showing a ‘Type 2’ existing street configuration with 11.5 m or above street roadway width, 2.35 m parking spaces on either side of the streets and two-way single traffic lanes of 3.4 m width to accommodate 50–60 km/h speed limit.

**Figure 7:**
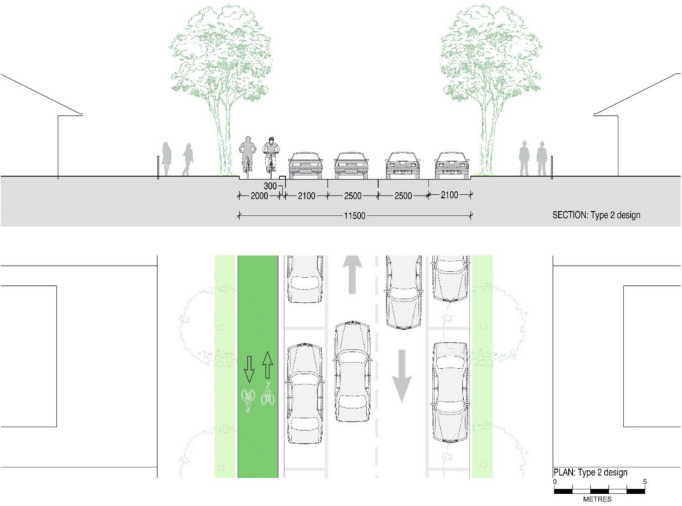
Section and plan drawings showing proposed retrofit ‘Design 2A’ for a reconfigured ‘Type 2’ street including a 2 m two-way cycling lane and 0.3 m wide physical barrier with similar roadway, but 2.1 m parking lanes on either side of the streets and two-way single traffic lanes of 2.5 m width to accommodate 40 km/h speed limit.

Figure 6 depicts an existing street configuration for the Type 2 streets we defined. This design layout is analogous to the Type 1 streets but comes with comparatively wide 3.4 m traffic lanes. These lanes can be reduced to 2.5 m with a reduced speed limit of 40 km/h [59], freeing up 1.8 m space to include a cycling lane. By reducing the 2.35 m wide parking spaces to the required minimum width for parking of 2.1 m, an additional 0.5 m space is freed up. Therefore, from Type 2 streets, we obtain 2.3 m space to include a 2 m wide two-way cycling lane with a 0.3 m wide physical separator as displayed in Figure 7. Design 2A is different from designs 1A and 1B as it allows cyclists to ride in both directions in the same street section.

These new street design layouts (Design 1A, 1B and 2A) optimise the use of the available street space with regards to providing safe cycling infrastructure while not requiring major revisions to existing street configurations.

### 3.3. Street Design Application to Study Road Network

In this initial study, we consider only a subset of the most prevalent local streets (Type 1 and Type 2), excluding narrower local streets, because the redesign of narrow streets requires the exclusion of facilities or substantial building works to include safe cycling. Additionally, new suburbs that are emerging are typically designed with wider streets that can have safe cycling added at the time of development. The application of new cycling designs to these streets can facilitate safe cycling while not impeding other traffic. To demonstrate this, we apply these designs to the road network and build two potential future design models. For the first Test Case, we selectively applied new designs to Type 1 and 2 streets and set the speed limits for all residential streets at 40 km/h.

In the second Test Case, we limited the speed reduction of 40 km/h speed limit just to the local streets with proposed cycle lane additions and maintained the existing 50 km/h speed limit for all other non-modified local streets. This approach assumes that cyclists are willing to take minor detours to use the new safer paths. One-way cycling lane designs are applied to Type 1 streets. Streets with less than a 10.5 m width were not considered for reconfiguration. Parking spaces were left in place to avoid inconveniencing residents, and street verges were left unchanged to avoid expense and disruption to services. This does not preclude future work on other possible scenarios with the remaining street typologies, for example, making narrow streets one-way or removing parking space to include safe cycling.

In the current work, designs 1A and 1B are selectively applied to the Type 1 local streets in the study area. Since these design options provide single direction cycling, we carefully selected streets to increase the continuity of paths through the cycling network. In addition, Design 2A, which is two-way cycling lane reconfiguration, is applied to Type 2 streets. However, modelling two-way cycling lanes in the Open Street Map (OSM) road network is a challenge due to modelling complexities.

Therefore, to mitigate this complexity and to include two-way cycling as in design 2A, we modelled one-way cycling lanes on each side street section in opposite directions. This application will allow cyclists to travel in both directions in the same street section. In this simulation software, parking lanes are modelled. In the simulation software, we have assumed parking spaces are present, but have not modelled cars entering to streets from parking lanes and vice versa, as the car parking condition is unaltered in all scenarios, and would introduce unnecessary complexity to the simulation.

### 3.4. Modelling Traffic Demand

The policies and initiatives around the ‘Living Locally’ concept in Melbourne aim to make it possible for residents to access necessities with less than a 20-min walk in the future [21]. This motivates our assumptions about an example suburb in Melbourne, its residents and the services these residents can reach on a bicycle. The case study based on these assumptions assumes the residents create a local traffic demand by using a mix of cars and bicycles on local streets and arterial roads for their daily trips to services available within the network. A typical Australian suburb consists of multiple main services (Destinations) including a few schools, restaurants, shopping centres, supermarkets, grocery shops, banks, post offices, parks, train stations, bus stops, aged care facilities, medical centres, pharmacies and a library. Therefore, the main local traffic is assumed to take place between these services (Destinations) and residences (Origins) trips within the local street network.

As most people use cars for travel purposes while few people walk and cycle (Statistics, 2017), the majority of the traffic demand used in the simulation are cars (80%) and a minority bicycle (20%). The local traffic demand (OD trips) for this study area is created based on the following assumptions about typical trips.

1. There is no impact from weather on cyclists’ mobility.
2. The residents in residential areas would cycle to nearby (less than 5 km) services (shopping centre, supermarkets, train station, schools, restaurants, post office, banks, parks and library).
3. Trips originate in residences in the living area and their destinations are the services within the same local network.
4. People go grocery shopping twice a week on average.
5. People see their doctor once a month on average.
6. People go to the park once a week on average.
7. On average, 2 people from 10 residences go to the train station every weekday during morning peak hours.
8. People go to the library, post office and banks once a week on average.
9. On average, 2 people will cycle to other services available in the network.

### 3.5. Traffic Simulation

#### Traffic Simulation Model

This study aims to explore the network efficiency in terms of speed, travel time and distance after the application of street designs for safe cycling. The AIMSUN traffic planning software is employed to simulate the network models. A representative sample of the residential road network of an Australian suburb is extracted from OSM, including a model of the traffic flows in the streets considered, based on current configurations. The AIMSUN simulator uses centroids to represent the origins and destinations (ODs) of the trips. To reflect the demand from residents, centroids were placed such that they connect to each intersection, representing the residents of a street. The demand was created by estimating the number of residents assumed to start a trip at their home and assigning them a destination centroid close to the pertinent service. Three OD matrices including car demand, bicycle demand and mixed demand (combination of car and bicycle traffic demand) were created for this experiment. The OD matrices were simulated for 4 h from 8 a.m. to 12 p.m. including morning rush hours. Based on the assumption about the residents’ daily travel behaviour, the car traffic OD matrix includes a total of 800 car trips and the bicycle matrix includes 200 bicycle trips. Using stochastic route choice, the simulator sends vehicles down the shortest path and revises the paths depending on congestion. A ‘microscopic simulation’ determines the travel times of the vehicles based on lane changing and car-following models.

## 4. Evaluation

### 4.1. Study Road Network

The local streets in Australian suburbs are generally organised as a grid with most streets running either West-East or North-South, and some cul-de-sac residential streets. Our study is based on the outer south-eastern suburb of Springvale, which is 22 km from Melbourne’s CBD. The road network of the study area mostly consists of local streets and only a few arterial roads. The current study is mainly focused on the cycling network optimisation of local streets. The suburb of Springvale includes major amenities such as a shopping centre, supermarkets, train station, schools, restaurants, post office, banks, parks, library, etc., which residents frequently access.

**Figure 8:**
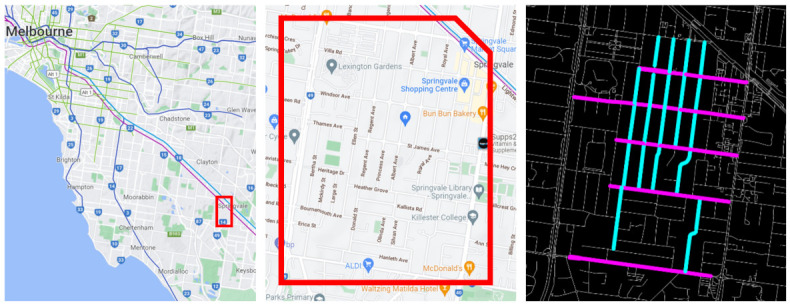
Location map showing the study area to the South-East of metropolitan Melbourne (**left**), the map of the study area next to Springvale Road (**middle**) and the street typology plan showing Type 1 streets (10.5 m wide) coloured cyan, and Type 2 streets (11.5 m wide) coloured magenta (**right**).

Having measured the average widths of the streets in the area using Near map, we found that Type 1 streets (10.5 m wide), shown in cyan colour, and Type 2 streets (11.5 m wide) in magenta colour in Figure 8 happen to run in West-East and North-South directions, respectively.

The road network of Springvale includes part of a cycling trail (Djerring Trail) which provides access to Springvale train station. We linked the network of cycling-enabled streets with the cycling trail for access to the train station. There is a trunk road (Springvale Road) in this study area with a reduced speed limit of 60 km/h as it runs through the Springvale city centre with main services on both sides of the road. In addition, this road network consists of primary, tertiary and unclassified roads. In this local street network, there are no bus stops.

#### 4.1.1. Baseline Network Model

The baseline network model represents the existing study area and the current vehicular behaviour. The baseline model does not contain any dedicated cycling lanes. Reflecting current reality, cyclists share the local street with the other road users. The simulation model was developed using mixed demand corresponding to the status quo. The current speed limit of 50 km/h for residential streets is applied to the corresponding streets in the network model. A Melbourne cycling study recorded a 22.7 km/h average speed for cyclists [40]. Therefore, for this study, the mean speed of bicycles was set to 20 km/h in all network models. Two scenarios of the baseline model were created by assuming travel demands with and without cyclists.

#### 4.1.2. Test Case 1: Selectively Reconfigured Cycling Network Model (40 km/h Speed Limit in Residential Streets)

The baseline model was modified to include cycling lane designs in the network within the study area. Designs 1A and 1B were applied to Type 1 streets and Design 2A to Type 2 streets (shown in Figure 8). The Type 2 local streets were modelled with separate one-way cycling lanes on each side as displayed in street design 2A. The Type 1 streets were designed with a one-way cycling lane for one side of the street as in design options 1A and 1B to represent the corresponding street in other directions for cycling continuity. The maximum speed of a residential street section has been changed to 40 km/h since the traffic lane width was reduced to accommodate the cycling lanes and a physical separator between the cycling lane and traffic lane. In our model, we restricted cyclists’ ability to enter residential streets without cycling lanes to simulate the notion that cyclists would not want to use mixed situations if offered dedicated lanes on streets nearby. Where no alternative path with separate cycling was available, cyclists were permitted to use residential streets with mixed traffic. As in the baseline and Test Case 1, car-only demand and mixed demand was simulated in two scenarios.

#### 4.1.3. Test Case 2: Selectively Reconfigured Cycling Network Model (Mixed Speed Limits in Residential Streets)

Test Case 2 was designed as an experiment to test the reduction of speeds to 40 km/h, limited to just streets with the proposed cycling lanes. Test Case 2 is a replication of Test Case 1 with the application of cycling lane designs to Type 1 and Type 2 streets. We set the speed limit to 40 km/h on the residential streets with cycling lanes, reflecting the need for speed reduction on narrower lanes. The remaining residential streets were left at the current 50 km/h, as their layouts remain unchanged. This test case caters for residents reluctant to accept reduced speed limits by minimising the number of streets they are applied to. The same OD demand (car only and mixed) as in the baseline model was assumed.

## 5. Results

In this section, we discuss the simulation results of each test case and compare them to those of the baseline model under four sections including model set-up statistics, variations of traffic measures of cars, and implications of cycling lanes on the speed with a higher traffic demand anticipated in the future. The test case models differ in the redesign of select streets but have equal traffic demand and other network parameters excluding the speed limit of residential streets. These scenarios were simulated many times with random seeds until we gained reliable average statistical measures. The average results were obtained after 20 replications of each scenario. The average speed, average travel time per trip and average distance travelled by a vehicle are used to analyse how cycling lanes in the road network impact the other road users.

### 5.1. Model Set-Up Statistics

AIMSUN uses the stochastic route choice method to assign routes to OD-demands, starting with the shortest path by distance, then revising the assignments every 15 min according to the travel times. Given the relatively low demand, the simulation shows the network to be in a free-flow state where cars travel at the speed limit or the desired speed assigned within a predefined range.

AIMSUN probabilistically releases the vehicles into the network during the simulation period and these vehicles leave the network once they have reached their destinations. The assumption of 800 car trips and 200 bicycle trips in 4 h (8 a.m. to 12 p.m.) is realistic for a low-density residential area with single- or double-storey residences. We observed that few cars and bicycles remain inside the network without completing their trips after simulations which amounts to around 1% of the trip completion rate. Consequently, the actual number of vehicles in the system varies compared to the specified demand. At least 99% of the vehicles have completed their trips in all scenarios, which ensures the results are comparable between scenarios. Table 1 lists the exact trip numbers and completion rates for the scenarios.

### 5.2. Variations of Traffic Measures of Cars

#### 5.2.1. Average Speed of a Car

**Figure 9:**
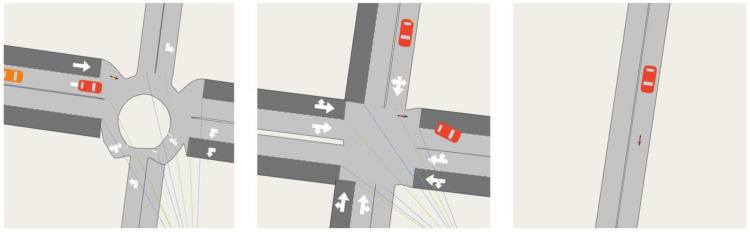
Street sections displaying interactions of bicycles and cars at a roundabout (**left**), intersection (**middle**) and at streets where space is shared for mixed traffic (**right**) during a simulation.

Table 2 summarises the key statistical traffic measures of the baseline model and two test cases. The current speed limit for residential streets is 50 km/h in most Australian cities, therefore this has been applied to our baseline model. Given the sparse population of vehicles, the average car speed we observe in the simulation is mostly free-flow, i.e., cars travel at the speed limit, apart from slowdowns caused by roundabouts and intersections as displayed in Figure 9. Simulation of car-only demand in the baseline model recorded the average speed of cars as 45.84 km/h. A simulation of cars with cyclists as a mixed demand within the baseline network model recorded a slightly lower average car speed of 45.5 km/h. The baseline model simulation results indicate only a slight speed reduction with given low traffic demand. Contrary to people’s perceptions, bicycles cause only an imperceptible delay on average, a result that has recently been corroborated by a study conducted in Portland, Oregon, USA, with a low speed limit of 25 mph (40 km/h) and low traffic demand. The results of statistical analysis (*t*-test and confidence interval) indicated that the maximum reduction of car speed is 1.6 km/h for specified shared streets [60].

In this road network, we applied the new street designs to add safe cycling lanes. Designs for Type 1 streets in our study area include a one-way cycling lane as shown in Figure 4 and Figure 5, and a design with a two-way cycling lane is introduced in Type 2 streets as displayed in Figure 7. The design which contains a two-way cycling lane ensures continuity for cyclists compared with one-way cycling lane designs.

According to our simulations, reducing speed limits and implementing dedicated safe cycling lanes has no significant impact on average travel times and average speed reduction of motorists. Contrary to popular opinion, the effects of including separate cycling lanes and reducing speed limits are relatively small. In Test Case 1 with the speed limit of 40 km/h and dedicated cycling lanes, the average speed of cars with and without cyclists is 40.95 km/h and 40.9 km/h, respectively. This suggests that the speed reduction compared to the baseline is due to the drop in speed limits. An overall 4.6 km/h drop in average speed was registered when we reduced the speed limit from 50 km/h to 40 km/h in Test Case 1, compared to the baseline average car speed (45.5 km/h) with mixed traffic simulation. Similar to Test Case 1, the average speed of cars in Test Case 2 for both simulations is approximately 42 km/h as displayed in Table 2. In Test Case 2, the overall speed reduction is 3.01 km/h compared with the baseline model. This reveals the effect of the cycling network on average speed is marginal. However, people travel slightly faster in Test Case 2 than Test Case 1, because in this case, 50 km/h was retained in streets with no dedicated cycling lanes. These results suggest that cyclists in the network with dedicated cycling lanes will not significantly reduce the speed of motor vehicles in the network.

#### 5.2.2. Average Travel Time of a Car

The increase in average travel time in Test Case 1 with cyclists on dedicated cycling lanes in the network is 5.65 s (about 7%) compared to the baseline mixed traffic demand simulation when cyclists share street space with cars. In Test Case 2, where we modified the speed limits to 40 km/h and 50 km/h, the increase in average travel time is even smaller than in Test Case 1 which is 2.89 s compared to the baseline model. The standard deviation for travel time per trip by car indicates that the travel time increase of a car trip is an average 8.26 s and 5.92 s in Test Case 1 and Test Case 2, respectively. This time variation suggests that the implementation of dedicated cycling lanes and reduced speed limits in residential streets contributes to less than 10 s delays per trip, which is negligible from a practical perspective.

#### 5.2.3. Average Distance Travelled by Car

The resulting average distance travelled by car is approximately 0.9 km for all scenarios as displayed in Table 2 regardless of the presence of dedicated cycling lanes. The trips vary between a maximum of 0.949 km and a minimum of 0.891 km in Test Case 1, while the longest and shortest trips for Test Case 2 vary between 0.934 km and 0.866 km. This indicates exclusive cycling lanes in road networks do not change the travel routes of the motorists. In the baseline model, the average distance travelled by car with and without cyclists in the network is similar (0.89 km). In addition, both simulations of Test Case 1 result in an average distance of 0.92 km, which represents a 3.3% increase compared with the baseline. However, in Test Case 2, we observe slightly fewer kilometres driven in the mixed demand simulation (0.9 km) compared with the car-only demand scenario (0.91 km). The observed average distance variations in Test Case 2 are due to the probabilistic nature of route assignment. Our study network is relatively small for all scenarios where, on average, people drive 900 m to reach the services in the network.

### 5.3. Implications of Cycling Lanes on Average Car Speed with Higher Traffic Demand 

In all three network models, the average speed of the cars is slightly lower in the mixed traffic demand simulation compared to the car-only demand simulations. In Test Cases 1 and 2, although cyclists use dedicated cycling lanes, cyclists and cars mix at roundabouts, intersections and with a few local streets where services are located as displayed in Figure 9. This behaviour reduces the average speed of cars marginally in mixed traffic simulations of all three scenarios even in free-flow conditions. In the baseline model, the average car speeds of both scenarios also have a slight difference, and the average car speed is a little higher when there are no cyclists in the network.

**Figure 10:**
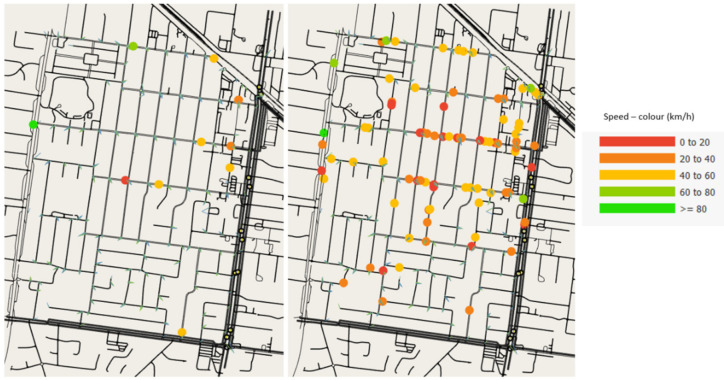
Cars and bicycles in the road network with current traffic demand (**left**) and stressed demand during a simulation (**right**).

These speed variations are minor in free-flow conditions. In addition to our testing based on relatively low traffic numbers reflecting existing conditions, we also ‘stressed’ the network with higher demands and recorded the average speed of cars assumed otherwise identical. With 9600 cars and 2400 cyclists in the network, we observed a significant reduction in the average speed of 5.15 km/h in the mixed demand simulation of the baseline. This result demonstrates the reduction of car speed when the network is congested while sharing street space with cyclists, as shown in Figure 10. The average car speed was also reduced in Test Cases 1 and 2, but only by 0.95 km/h and 1.53 km/h, respectively, a minor increase compared to the baseline. The relatively higher speeds show that if cyclists use dedicated cycling lanes and share the street with cars only when absolutely necessary (e.g., at intersections), the impact of cycling on car speed is negligible.

### 5.4. Trip Variations of Cyclists

According to the simulations, the average cycling speed is approximately 19 km/h across all three scenarios. We did not model separate cycling lanes at the intersections and roundabouts to avoid potentially prohibitive space and cost implications. In Test Cases 1 and 2, we deliberately restricted cyclist access to streets without dedicated cycling lanes where alternatives exist. This reflects the assumption that cyclists would choose to take slight detours to use cycling lanes where possible. As a consequence of the assumption that cyclists would prefer safe lanes to shortest paths, the average travel distance and time are clearly longer in Test Cases 1 and 2 compared to the baseline. In all three scenarios, cyclists interact with cars at roundabouts and intersections, which necessarily has an impact on the average cycling speeds, too. While we modelled mixed traffic for this study, other safe design approaches for roundabouts such as those described by Cumming could be integrated into our model [61]. Longer journeys are likely to incorporate more junctions in a trip, which is the likely cause of the very minor decrease in cycling speed (19.67 km/h in the baseline and 19.3 km/h in Test Case 2). The average travel time of a cyclist increases by 35 to 45 s in both scenarios with dedicated cycling lanes, compared to the baseline. The average distance travelled by a cyclist in the baseline model is 0.93 km, but 1.11 km and 1.15 km in Test Cases 1 and 2.

## 6. Discussion

Most residents assume the implementation of cycling lanes on residential streets will negatively affect their neighbourhood [62]. Cycling infrastructure is also assumed to reduce on-street parking and contribute to a decrease in property values [62]. Consequently, most Australian cycling lanes are painted-on lanes without physical separation that allow parking, creating a need for cyclists to mix with traffic to dodge parked cars. To respond to the complex social, political and built environment conditions, we did not reduce existing parking spaces while managing to introduce safe cycling paths, by narrowing car-parking lane widths and traffic lane widths. This necessitated a reduction in the speed limit to 40 km/h, which we believe is a desirable measure to improve safety regardless of cycling spaces. As tighter speed limits also often encounter resistance, we included an alternative test case where unaltered streets remain at 50 km/h to establish the impact of reducing speeds only where the space limitations mandate it.

Given the low local traffic load appropriate for low-density suburbs, the simulation has most vehicles travelling at free-flow speeds. In spite of the 50 km/h speed limit in the baseline model, reflecting current conditions, the simulations resulted in an average car speed of 45 km/h. The trips are primarily routed through residential streets, but one trunk road with a speed limit of 60 km/h traverses it in the South-North direction and two primary roads with 80 km/h and 60 km/h speed limits. While intersections and roundabouts decrease the average speeds recorded, the higher speed limits on trunk and primary roads augment them. The simulator assigns speeds probabilistically to account for people who tend to speed as well as slower drivers. The default speed probability distributions of the simulator were retained for all trials, as were the parameter values of slowdowns at turns. The microsimulation model made assumptions about driver behaviour around other cars and cyclists. This represents actual traffic observations, such as motorists tending to reduce speed when overtaking cyclists and moving further into the opposite lane to keep their distance from the cyclists [63]. 

In this study network, bus stops were not a contributing factor to the model as we exclude application of cycling lanes on arterial roads where some are present. The implications of car and cyclist stoppage due to the bus stops would need to be included in the model in areas that have bus stops on minor roads, or where arterial roads were included.

Many studies have found that the inclination of roads, traffic conditions, number of intersections, bicycle lane infrastructure, age, gender, the purpose of trip and biomechanics have an impact on the speed of cyclists [64,65,66,67]. In our study area, most residential streets have flat surfaces, many roundabouts and intersections. The simulation results indicate that the travel time of cyclists increases marginally due to the detours they made to follow cycling lanes. While the ideal scenario for cyclists would be to not increase travel distances at all, we believe the minimal detours for physically separated safe cycle lanes would be a trade-off which most cyclists would be willing to make. These small detours would have even less impact on those using e-bikes. 

The study area examined has low density development and low levels of walking activity without designated pedestrian crossings; we therefore did not include pedestrians in the model. In denser urban development with more mixed uses and higher foot-traffic, it would be important to incorporate the pedestrian-agents to observe the impact on network efficiency.

## 7. Validity of Simulation

The validity of our findings hinges on the authenticity of the traffic network and car-following models in the simulator. The Springvale network extracted from OSM was used to model the traffic behaviour assumptions that go with each street design. The baseline model uses the OSM lanes without change, allowing cars and bicycles. The redesigned streets use separate lanes for each type of vehicle and disallow cycling on streets without cycling lanes. Microsimulations determine the speeds of vehicles by modelling the interactions between vehicles and the interaction with infrastructure. Vehicles that cross each other’s paths may slow each other down when arriving at an intersection at the same time. Vehicles are slowed down by tight turns. The default parameters in AIMSUN were used for these interactions. Possible inaccuracies in the model of these interactions would likely affect the scenarios chosen in equal measure.

The choice of Springvale as a representative suburb could be challenged, as some other neighbourhoods in Melbourne—or indeed other major cities—may have predominantly narrower or wider streets, or different lane configurations. Additionally, the effects of transit traffic (trips starting and ending outside Springvale) on congestion of the network have been omitted.

Transit traffic is expected to affect mostly trunk roads and arterials, but possibly also residential streets. Verifying the effects of this requires a much larger network, which is beyond the scope of this study.

## 8. Future Work

This initial study has confined the selective street reconfiguration to streets with a certain width. Narrower streets necessitate a different cycling-inclusive design, and wider streets have a potential to include wider lanes or space for more types of functionality such as angle parking. Including designs that suit (almost) all residential streets provide an opportunity for a network optimisation that would benefit vehicle and bicycle traffic. In further work, we will devise and analyse the potential designs for a comprehensive number of current street widths and layouts. Designs with one-way streets for vehicles and/or cyclists enhance the possibilities for a continuous cycling network. An automated method for redesigning and optimising the network is also part of our plans for future work, as is the development of suitable network appraisal measures.

Once these extensions have been added, it will be possible to simulate a larger part of the street network, which will add insights into the impact of trips over longer distances, such as commutes.

## 9. Conclusions

While cycling is a sustainable transportation mode with many health, economic and environmental benefits, participation is at a very low level in Australia. Many major cities consist of a considerable amount of cycling infrastructure, i.e., generally painted-on bike lanes on streets, which is not functional in terms of safety and continuity. This study reinforces that modifying legacy infrastructure by adding safe, physically separated cycling lanes in residential streets with reduced speed limits has a minor effect on existing traffic conditions. The results show that the effect of the application of bike lane designs and reduction of speed limits in this study area on the average speed of cars, time and distance travelled are insignificant from a practical perspective. The delay incurred by drivers as a consequence of including safe cycling lanes in some residential streets is less than an extra 10 s per kilometre. The results show that the safe cycling network modelled in the test cases introduced only a small increase in travel time for cyclists due to detours arising from the selective application of safe cycling lanes. Many cyclists are likely to consider this a small trade-off for their safety, the extent of which is an area worthy of further study. There are also opportunities for further optimisation of cycle lanes and street designs.

The new designs applied in the study site network greatly improve safety by providing physically separated cycling lanes while keeping all existing car parking spaces and avoiding major street modifications and traffic delays. Street reconfigurations with minimal changes keep 90% of the bicycle trips within a safe cycling network. Hence, this is a favourable option for local councils to introduce healthy, active transport through inclusion of safe cycling networks in residential areas as it requires relatively low-cost street modifications, and is not likely to cause major political controversy. Based on this pilot study, further work can automate the modelling process for more complex and relatively large residential areas containing diverse street width typologies to include safe bike lanes with design alternatives to provide the most efficient cycling routes within the network. This could involve reconfiguring two-way streets into one-way streets, or two-way streets without parking to include safe cycling.

This pilot study asked the question: “Can the roadways of local streets be reconfigured to include safe cycling lanes?”. We demonstrated that, yes, it is possible to retrofit local streets with cycle lanes through the adjustment of speed limits and reduction of parking and street lane widths. We also asked the question: “Do safe bike lanes really slow down cars?”. The answer, based on this traffic model of a representative suburb of Melbourne is—no, not to any consequential degree. 

With the growing need to increase levels of active travel for significant community health benefits, the need to transition to low-carbon mobility and reduced vehicular emission impact on the environment, the development of safe cycling infrastructure is critical in increasing the cycling uptake in Australia. Therefore, the approach described in this study has great potential to be beneficial for policymakers, urban designers and planners, traffic engineers and the wider community.

## Figures and Tables

**Table 1:** Model set-up.

	Baseline Scenario		Selectively Reconfigured Cycling Network Scenario (40 km/h)		Selectively Reconfigured Cycling Network Scenario (40 km/h and 50 km/h)	
	Car Demand	Mixed Demand	Car Demand	Mixed Demand	Car Demand	Mixed Demand
Cars~	800	800	800	800	800	800
Completion rate	99.54%	99.44%	99.51%	99.45%	99.41%	99.50%
Bicycles~		200		200		200
Completion rate		99.31%		98.94%		99.05%

**Table 2:** Summary of simulation results.

	Baseline Model (50 km/h)		Test Case 1: Selectively Reconfigured Model (40 km/h)		Test Case 2: Selectively Reconfigured Model (40 km/h and 50 km/h)	
	Car Demand Scenario	Mixed Demand Scenario	Car Demand Scenario	Mixed Demand Scenario	Car Demand Scenario	Mixed Demand Scenario
Speed of Car (km/h)	45.84	45.5	40.95	40.9	42.68	42.49
Average Distance Travelled by a Car (km)	0.89	0.89	0.92	0.92	0.91	0.90
Average Travel Time by a Car (seconds)	72.32	76.15	82.01	81.80	78.73	79.04
Delay Time per Car per km (seconds)	11.04	12.01	9.18	9.38	10.18	10.48
Speed of Bicycle(km/h)		19.67		19.57		19.3
Average Distance Travelled by Bicycle (km)		0.93		1.11		1.15
Average Travel Time by a Bicycle (seconds)		184.88		220.44		231.31
Speed of Car (km/h) Stressed demand)	45.1	40.35	40.41	39.95	41.84	40.96

## Data Availability

The road network models (.ang files) that produced simulation results in this study are available at https://github.com/Cycling-Project/Cycling-Network-Models, (accessed on 14 February 2022).
